# Mediating effect of job crafting dimensions on influence of burnout at self-efficacy and performance: revisiting health-impairment process of JD-R theory in public administration

**DOI:** 10.3389/fpsyg.2023.1137012

**Published:** 2023-04-28

**Authors:** Ana Martínez-Díaz, Pedro Antonio Díaz-Fúnez, Carmen María Salvador-Ferrer, Brizeida Raquel Hernández-Sánchez, José Carlos Sánchez-García, Miguel Ángel Mañas-Rodríguez

**Affiliations:** ^1^IPTORA Research Team, University of Almería, Almería, Spain; ^2^IPTORA Research Team, Department of Psychology, University of Almería, Almería, Spain; ^3^Department of Psychology, University of Salamanca, Salamanca, Spain

**Keywords:** burnout, job-crafting, self-efficacy, performance, public administration

## Abstract

**Introduction:**

In recent years, job crafting has greatly interested Work and Organizational Psychology. Different research studies have shown its positive impact on people and organizational performance. However, it knows little about the differential effect of the two dimensions that make up this variable (prevention-focused and promotion-focused) and its role in the health-impairment spiral process of the job demand-resources theory (JD-R).

**Method:**

This research aims to analyze the mediating effect of the different dimensions of job crafting on the influence of burnout on performance and self-efficacy in the workplace. The study used a sample of 339 administrative employees of a university.

**Results:**

The results indicate that promotion-focused job crafting is a mediating variable in the relationship between the influence of burnout on performance and self-efficacy. Unexpectedly, prevention-focused job crafting does not have this mediating role in the same relationship.

**Discussion:**

These findings confirm the adverse impact of burnout on personal and organizational improvement, while showing the absence of prevention/protection responses of employees when they are burned out. The theoretical and practical implications show an advance in knowledge about the process of health deterioration and about the spiral of health deterioration in the JD-R theory.

## 1. Introduction

According to the World Health Organization (WHO), work is essential for people’s well-being (World Health Organization, 1976). This means placing occupational health strategies as the key to research in the promotion of well-being in the workplace. The International Labor Organization (International Labour Organization, 2009) and the WHO define occupational health as the promotion of physical, mental, and social well-being of employees. These factors, added to the increase in resources such as team climate, and the reduction of job demands, are crucial elements in organizations for workers to feel self-efficacious and perform successfully in their work (Mañas-Rodríguez et al., 1999; Pecino et al., 2019; Martínez-Díaz et al., 2020).

**Figure 1:**
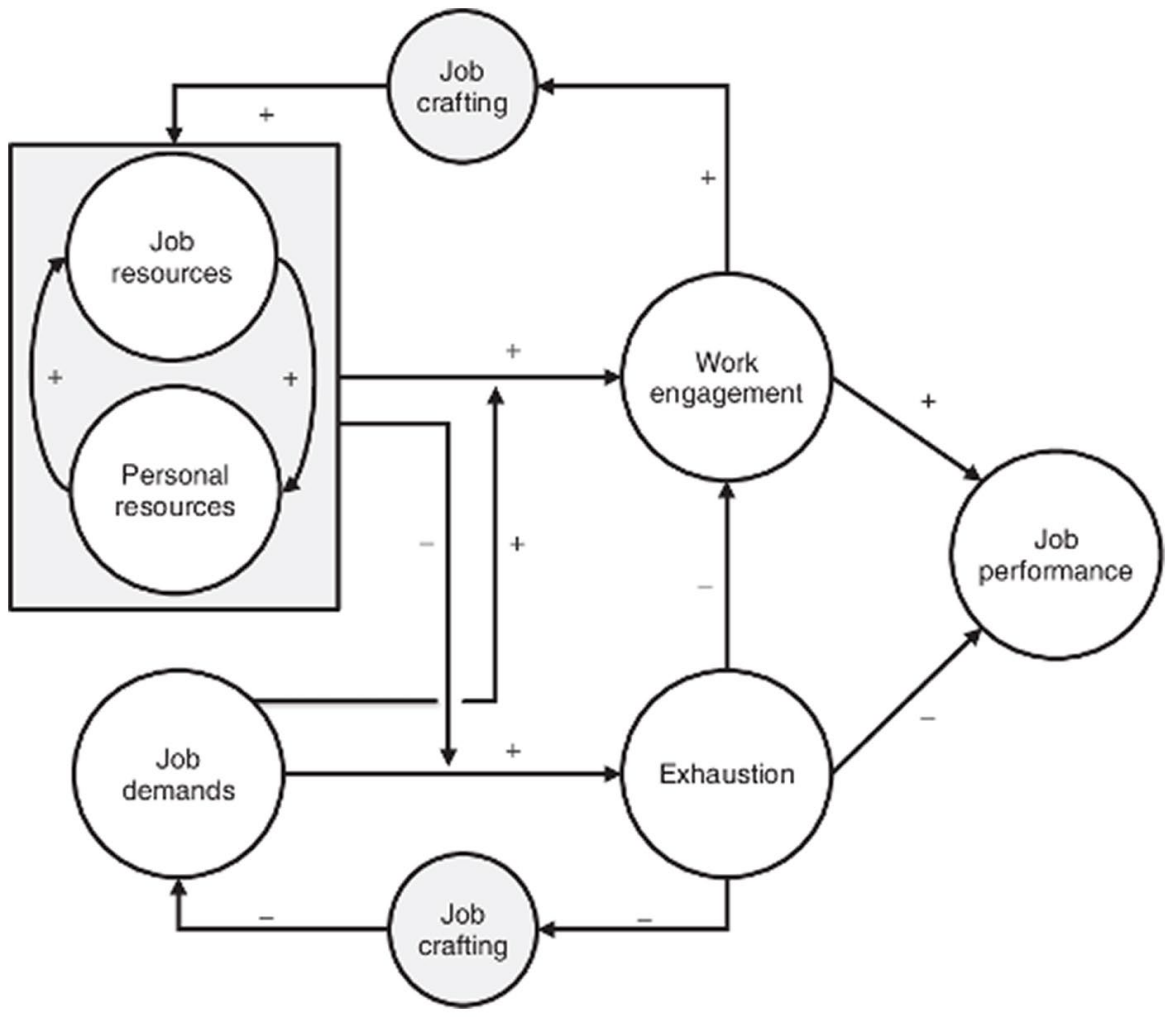
JD-R theory. Adapted from Bakker and Demerouti (2013).

The theoretical framework that has received the most attention in studies on the promotion of employees’ well-being is the job demands-resources theory (JD-R theory; Bakker and Demerouti, 2013). According to this theory, job characteristics can be organized into demands and resources (see Figure 1; Tims et al., 2012).

Bakker and Demerouti (2014) define job demands as the physical, psychological, organizational, or social aspects of work that require sustained effort and entail both physiological and psychological costs. These are the main threat to the promotion of occupational health and the well-being of employees. Instead, the job resources are described by Bakker and Demerouti (2017) as the physical, psychological, organizational, or social aspects existing in the work context that can reduce these demands and the physiological and psychological costs associated with the work environment. The JD-R theory states that the dynamic relationship between job demands and resources triggers started a health impairment or a motivational process (Bakker et al., 2022).

This research focuses on how the health impairment process influences the worker’s responses at the organizational level. Faced with a negative context, workers act by changing aspects of their job, choosing tasks, negotiating work content, or assigning new meanings to their work (Albrecht et al., 2015). This adaptation of the position by the worker to fit the work context is called job crafting (Wrzesniewski and Dutton, 2001). This term is used in the JD-R theory as an explanatory variable by the spiral gain and impairment process (Bakker and Demerouti, 2013). This theory proposes that job crafting is a proactive behavior defined as “*the changes that workers make to align their demands and personal job resources with their own needs and capabilities*” (Tims and Bakker, 2010, p. 3).

One aspect to consider in job crafting is the need for the worker to present three individual characteristics (Wrzesniewski and Dutton, 2001). First, there must be active participation on the part of the worker to control certain aspects of the work avoid negative consequences. Second, workers must be motivated to change elements of their job to have a more positive view of themselves. Thirdly, it must allow an increase in social involvement since there is a perception of connection with others. But little is known about the response produced in job crafting when the organizational context is negative.

Research has proposed an evolution in understanding the concept of job crafting. Initially, Petrou et al. (2012) defended a global conceptualization of it, where people want to modify certain aspects of their work to create conditions where they can work healthier and more motivated. Tims et al. (2012) proposed the existence of four different behaviors to carry out these changes: (a) increase structural job resources; (b) increase social job resources; (c) increase challenging job demands, and (d) reduce job demands that are an obstacle. One of the most recent approaches to job crafting is developed by Lichtenthaler and Fischbach (2019). These authors distinguished between two job crafting processes: prevention-focused and promotion-focused.

Prevention-focused job crafting could be defined as employees’ protecting behaviors, avoiding obstacles and end demands, in which they anticipate discomfort from non-compliance, stagnation, difficulties, and loss of energy, health, or safety. On the other hand, promotion-focused job crafting encompasses the changes through which workers achieve positive end states, anticipate achievement, learning, and growth, and gain exciting tasks, social relationships, and other motivating aspects of work. Integrating these two concepts with the typology of job crafting behaviors proposed by Tims et al. (2012), prevention-focused job crafting will be related to decreasing obstacle job demands and the impairment health process. Promotion-focused job crafting will be related to increasing structural job resources, growth of social job resources, and a rise in challenging job demands.

The distinction between prevention-focused and promotion-focused job crafting is of great research interest, as its determinants will influence differently depending on the distribution of positive or negative emotions in the work context (Dubbelt et al., 2019; Lazazzara et al., 2020). Authors such as Singh and Singh (2018) suggest that employee perceptions are among the most determining factors of job crafting. The JD-R theory confirms that a worker with negative emotions can modify his job in two ways: increasing the resources available to deal with them or reducing the influence of these emotions (Bakker and Demerouti, 2017). Changes in their job position would largely determine that the demands would facilitate goal achievement, with desirable consequences for both the worker and the organization (Tims et al., 2021). These are called challenge demands by LePine et al. (2005). Conversely, some demands are perceived by employees as hindering and may be detrimental to work readiness behaviors. These demands are called hindrance demands.

One emotional consequence of obstacles that has been shown to influence job crafting is burnout (Bakker and de Vries, 2021). However, researchers do not clarify how this variable affects the prevention-focused and promotion-focused dimensions of job crafting in the impairment health process. Research findings suggest that prevention-focused job crafting behaviors could increase in the face of the employee’s perception of burnout (Zhang and Parker, 2019; Singh and Rajput, 2021). In contrast, promotion-focused job crafting behaviors would decrease (Lichtenthaler and Fischbach, 2019). At this point, it is worth asking what effect burnout will have on the prevention and promotion dimensions of job crafting on employees and their performance. JD-R theory has provided an answer to this question. This theoretical framework proposes that the effects of job crafting will have a direct impact on the gain (work engagement-job crafting-new resources influence) or the impairment (exhaustion-job crafting-new demands influence) spirals in the work context (Bakker and Demerouti, 2013). Tims et al. (2014) researched the consequences of job crafting and found that these behaviors have a high level of influence on the employee’s resources, specifically on the perception of self-efficacy.

Although the JD-R theory does not directly pose this, some studies have shown the influence of job crafting on organizational objectives such as performance. This effect was in research such as that developed by Tims et al. (2014) or the study by Miraglia et al. (2017) on 465 public administration workers. Their results confirm the positive influence on the performance of workers over time.

The review of burnout has also revealed the weight of this requirement on performance and self-efficacy perceptions, with a negative sign. For example, Hosseini et al. (2017) showed a negative influence between burnout and nurses’ performance, highlighting that high burnout could drastically reduce performance. In another research, Aftab et al. (2012) indicate a statistically significant and negative relationship between these two variables assessed in a medical worker group.

Other theories that explain the effect of burnout on the rest of the study variables are the Yerkes–Dodson theory (1908) and the conservation resources theory (Hobfoll, 1989). In these theories, the equity hypothesis (Taris et al., 2002) determines that an unbalanced perception of job demands reduces well-being. However, the degree of burnout defines the balance level of a workplace context, and its effect on job crafting employees’ responses, due to the sensitivity to emotional events (Baumeister et al., 2001). In this sense, the increase in burnout employees’ perceptions would also mean an increase in their level of prevention job crafting behaviors and a decrease in promotion job crafting intentions acting as a challenging demand.

As we have seen, there is evidence linking burnout with job crafting and of both variables on the perception of self-efficacy and performance, but with a positive impact in the case of job crafting and a negative impact concerning burnout. The JD-R theory supports both influences. But this theory does not propose a direct effect of job crafting on performance. These effects would use different pathways: the positive ones would come from the motivational process and the negative ones from the health deterioration process.

**Figure 2:**
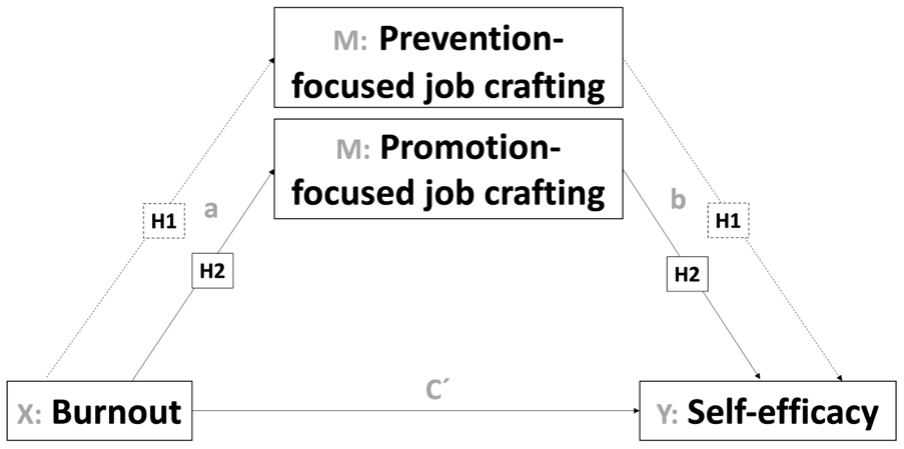
Mediation model of job crafting’s dimensions in the influence of burnout on self-efficacy.

**Figure 3:**
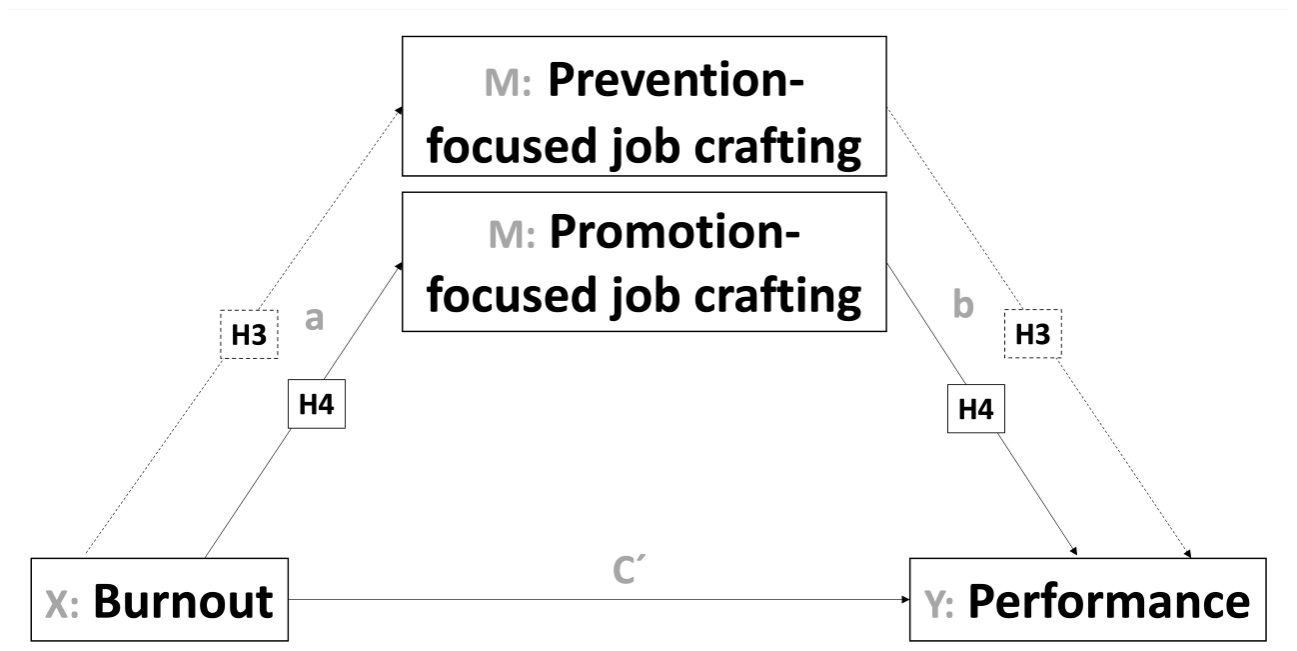
Mediation model of job crafting’s dimensions in the influence of burnout on performance.

Thus, the objective of this paper focuses on analyzing the role of two different types of job crafting as mediators in the influence of burnout on the perception of self-efficacy and performance. The health impairment process of the JD-R theory (Bakker and Demerouti, 2013) and the approaches of Lichtenthaler and Fischbach (2019) are a reference, from which the following hypotheses are proposed (see Figures 2, 3).

*H1*: Burnout will have a positive and significant effect on prevention-focused job crafting. Prevention-focused job crafting will act as a mediating variable in the influence of burnout on self-efficacy.

*H2*: Burnout will negatively and significantly influence promotion-focused job crafting. Promotion-focused job crafting will act as a mediate of the influence of burnout on self-efficacy.

*H3*: Burnout will have a positive and significant effect on prevention-focused job crafting. Prevention-focused job crafting will act as a mediator in the influence of burnout on performance.

*H4*: Burnout will have a negative and significant influence on promotion-focused job crafting. Promotion-focused will be a mediator in the influence of burnout on performance.

In this research proposal, the novelty of this contribution concerns other investigations concerning resources in the negative spiral posed by the deterioration of the health process in the theory of resources and labor demands (Bakker and Demerouti, 2007). Within this process, the present study tries to clarify three aspects. First, it delves into the influence of burnout on the dimensions of job crafting (prevention and promotion), which will allow increasing knowledge about the antecedents related to the reduction of these employee behaviors. Secondly, it aims to broaden the range of backgrounds in job crafting. Most previous publications have focused their interest on individual traits, such as personality, or ones, such as the exhaustion of the JD-R theory (Hosseini et al., 2017) as determinants of these behaviors in employees. This study proposes as an antecedent a state generated by the configuration of work characteristics, such as the worker’s perception of being burned out at work, thus giving a broader perspective to the process of health deterioration. Third, the study aims to demonstrate the mediating influence exerted by the job crafting dimensions between a negative state of the work environment (burnout) and two results in the organization: the intention of performance by the worker and their perception of self-efficacy (Lichtenthaler and Fischbach, 2019). In other words, it is proposed to analyze whether job crafting responses will be one of how burnout influences individual performance and self-perception of employee efficacy, thus completing the spiral of health deterioration posed in the article. Bakker and Demerouti model (2007).

## 2. Materials and methods

### 2.1. Participants and procedure

In this descriptive study, data were collected through online questionnaires. In total, 402 public employees were invited to participate, who were distributed in 33 work teams with an average unit size of 18.08 (SD = 10.86). University Bioethics Committee approval was obtained for this study (UALBIO2018/002).

All the questionnaires collected, 339 (84.32%) were correctly completed and could be included in the analysis. Age was distributed within four intervals (from 26 to 35 years = 1.8%, 36 to 45 = 14.1%, 46 to 55 = 64%, and 56 or older = 20.1%). Regarding sex, 52% were men, and 48% were women. The level of education was distributed in these categories: Elementary school (10.3%), higher education (23%), College (63.4%), and master’s degree/Ph.D. (14.3%).

### 2.2. Instruments

#### 2.2.1. Prevention-focused and promotion-focused job crafting

Prevention-focused and promotion-focused job *crafting* was measured using the Spanish adaptation of the Job Crafting Scale made by Bakker et al. (2018). Prevention-focused dimension comprises six items (i.e., I make sure that my work is mentally less intense), promotion-focused dimension comprises fifteen items (i.e., I try to develop my abilities). Response options are delivered on a Likert scale ranging from 1 (never) to 7 (always), with higher scores indicating a higher level of job crafting. Prevention-focused scale obtained Cronbach’s alpha reliabilities of 0.89. Promotion-focused scale achieved Cronbach’s alpha reliabilities of 0.80.

#### 2.2.2. Burnout

Burnout was evaluated using the Spanish adaptation (Gil-Monte and Moreno-Jiménez, 2005) of the Maslach Burnout Inventory (MBI) developed by Maslach and Jackson (1981). This tool has three underlying dimensions: Exhaustion, which is composed of three items (i.e., I feel emotionally drained from my work); cynicism, composed of seven items (i.e., I have become less enthusiastic about my work); and efficacy, which consists of three items (i.e., I can effectively solve the problems that arise in my study/work). All items are scored on a 5-point frequency rating scale ranging from 1 (strongly disagree) to 5 (strongly agree). High scores on exhaustion and cynicism and low scores on efficacy are indicative of burnout (i.e., All efficacy items are reversibly scored). The internal consistency (Cronbach’s α) of the scale was 0.80.

#### 2.2.3. Self-efficacy

Self-efficacy was measured using the Spanish adaptation (León-Pérez et al., 2017) of the Psychological Capital Questionary (PCQ12) developed by Luthans et al. (2007). Self-efficacy comprises three items (i.e., I think I would represent my work group well in meetings with management). All items are scored on a 6-point frequency rating scale ranging from 1 (strongly disagree) to 6 (strongly agree). The internal consistency of the scale was 0.77.

#### 2.2.4. Performance

Performance was measured using the dimension in the Work Unit Performance Scale by Goodman and Svyantek (1999). The scale consists of three items that analyze actions in formal job descriptions and increase organizational effectiveness (i.e., “I willingly attend functions not required by the organization but help in its overall image”). Participants responded on a seven-point scale ranging from 1 (strongly disagree) to 7 (strongly agree). The internal consistency of the scale was 0.91.

#### 2.2.5. Control variables

Because the evaluation of the work context is sensitive to the sex and age of the employees (Bertolino et al., 2011), we control for these two demographic characteristics. A dichotomy scale (“Woman versus man”) was used for gender and a Likert scale with five categories (1: “18–25 years,” 2: “26–35 years,” 3: “36–45 years,” 4: “46–55 years,” and 5: “56 years or more”) to measure age.

### 2.3. Statistical analysis

Data were analyzed using IBM SPSS 27. After computing descriptive data, Cronbach’s alphas, and zero-order relationships between all constructs, mediation, and moderation analyses were conducted (see Figures 2, 3). Following the recommendations of Cristea et al. (2013), a multi-step mediation analysis was used to test whether the effect of burnout on self-efficacy and performance is mediated by prevention-focused job crafting and promotion-focused job crafting. Mediation analyses were conducted to estimate direct and indirect influence using the non-parametric bootstrapping procedure in the PROCESS package. The suggestion of Hayes (2017) was followed by conducting a multi-step mediation analysis to find the mediation effect (Model 4 in PROCESS).

Indirect and conditional influences were deemed significant if the 95% bias-corrected (BC) bootstrap confidence intervals (CI) based on 10,000 samples did not include. The fully standardized indirect effect (ab_cs_) was used to calculate mediation effect sizes, with 95% baseline confidence intervals for BC (Hayes and Matthes, 2009; Preacher and Kelley, 2011). This measurement is based on the product of the betas for routes a and b, which provides us with the expected change in the dependent variable (i.e., self-efficacy or performance) for each unit in which it varies in the predictor variable (i.e., burnout) indirectly through the mediator (i.e., prevention-focused, or promotion-focused job crafting).

## 3. Results

The results structure is two sections: First, present the descriptive results and correlations between the variables included in this study. Second, the results of the regression models where the influences of the prevention-focused job crafting and the promotion-focused job crafting on self-efficacy and performance are tested, in this order.

The descriptive data and the correlations between the study variables are provided in Table 1. The mean scores obtained have been prevention-focused job crafting (3.45), promotion-focused job crafting (4.46), burnout (2.08), self-efficacy (4.40), and performance (4.78). Except for the mean burnout scores, all the others exceed the mean value of the scale, being this 3 (burnout), 3.5 (self-efficacy), 4 (promotion-focused job crafting), 4 (prevention-focused job crafting), and 4 (performance).

**Table 1:** The means, standard deviations, and correlations between variables.

	*M*	SD	2	3	4	5	6	7
1. Burnout	2.08	0.69	−0.165**	−0.256**	−0.484**	−0.457**	–	0.004
2. Self-efficacy	4.40	1.01		0.457**	0.604**	0.310**	–	−0.023
3. Promotion focuses JC	4.46	0.89			0.591**	0.391**	–	−0.011
4. Prevention focuses JC	3.45	0.82				0.506**	–	−0.002
5. Performance	4.78	1.04					–	0.033
6. Gender	–	–						–
7. Age	4.10	1.00						

** *p* < 0.001; JC, Job crafting.

Regarding the correlation results, prevention-focused job crafting indicates a significant positive correlation with performance (0.506**).The promotion-focused job crafting also shows a significant and positive correlation with prevention-focused job crafting (0.591**) and performance (0.391**). Meanwhile, burnout presents a significant and negative correlation with self-efficacy (−0.165**), prevention-focused job crafting (−0.484**), promotion-focused job crafting (−0.256**), and performance (−0.457**). For its part, self-efficacy presents a significant and positive correlation with prevention-focused job crafting (0.604**), promotion-focused job crafting (0.457**), and performance (0.310**). The relationship between control variables and burnout, promotion focused job crafting, prevention focused job crafting, performance and self-efficacy were not significant.

Table 2 shows the multi-step mediation analysis of job crafting prevention behavior on the influence of burnout on self-efficacy. As indicated by the data from regression 1 X= > M (a), the burnout variable does not have a significant influence on the mediating variable prevention-focused job crafting (*B* = 0.024, *SE* = 0.101; *t* = −0.236, *p* = 0.813).

**Table 2:** Analysis of prevention-focuses job crafting mediation on the influence of burnout on self-efficacy (simple mediation model for prevention-focuses job crafting).

**Regression 1 X= > M (a)**				
Predictor	Outcome = M (prevention-focused job crafting)			
	*B*	*SE*	*t*	*p*
X (burnout)	0.024	0.101	−0.236	0.813
Constant	0.084	0.068	0.121	0.903
**Regression 2 X, M= > Y (c′ & b)**				
Predictor	Outcome = Y (self-efficacy)			
	*B*	*SE*	*t*	*p*
X (burnout)	−0.239	0.078	−3.057	0.002
M (prevention-focused job crafting)	0.091	0.042	2.155	0.031
Constant	4.402	0.053	82.082	0.000
**Regression 3 total effect (c)**				
Predictor	Outcome = Y (self-efficacy)			
	*B*	*SE*	*t*	*p*
X (burnout)	−0.246	0.078	−3.068	0.002
Constant	4.403	0.053	81.658	0.000

B, Beta; SE, Standard error; Bootstrap sample size = 10,000.

Table 3 shows the multi-step mediation analysis of job crafting promotion behavior in the influence of burnout on self-efficacy. In regression 1 X= > M (a), burnout shows a significant effect on the mediating variable promotion-focused job crafting (*B* = −0.337, *SE* = 0.069, *t* = −4.865 *p* < 0.01). Regression 2 X, M= > Y (c′ & b) shows a complete mediation of the promotion-focused job crafting on the influence of burnout on self-efficacy. The independent variable becomes non-significant when the mediating variable is included in equation regression. Regression 3 total effect shows that the total influence of burnout on self-efficacy was significant and negative (*B* = −0.241, *SE* = 0.078, *t* = −3.068; *p* = 0.002).

**Table 3:** Analysis of promotion-focuses job crafting mediation on the influence of burnout on self-efficacy (simple mediation model for promotion-focuses job crafting).

**Regression 1 X= > M (a)**				
Predictor	Outcome = M (promotion-focuses job crafting)			
	*B*	*SE*	*t*	*p*
X (burnout)	−0.337	0.069	−4.865	0.000
Constant	4.426	0.047	93.291	0.000
**Regression 2 X, M= > Y (c′ & b)**				
Predictor	Outcome = Y (self-efficacy)			
	*B*	*SE*	*t*	*p*
X (burnout)	−0.075	0.073	−1.020	0.308
M (promotion-focused job crafting)	0.494	0.055	8.857	0.000
Constant	2.214	0.251	8.793	0.000
**Regression 3 total effect (c)**				
Predictor	Outcome = Y (self-efficacy)			
	*B*	*SE*	*t*	*p*
X (burnout)	−0.241	0.078	−3.068	0.002
Constant	4.403	0.053	81.658	0.000

B, Beta; SE, Standard error; Bootstrap sample size = 10,000.

Table 4 shows the multi-step mediation analyses. We analyzed the influence of burnout on performance, including the effect of prevention-focused job crafting as a mediator. In regression 1 X => M (a), the variable burnout shows no significant influence on the mediating variable prevention-focused job crafting (*B* = 0.024, *SE* = 0.101; *t* = −0.236, *p* = 0.813).

**Table 4:** Analysis of prevention-focuses job crafting mediation on the influence of burnout on performance (simple mediation model for prevention-focuses job crafting).

**Regression 1 X= > M (a)**				
Predictor	Outcome = M (prevention-focused job crafting)			
	*B*	*SE*	*t*	*p*
X (burnout)	0.024	0.101	−0.236	0.813
Constant	0.084	0.068	0.121	0.903
**Regression 2 X, M= > Y (c′ & b)**				
Predictor	Outcome = Y (performance)			
	*B*	*SE*	*t*	*p*
X (burnout)	−0.680	0.075	−9.074	0.000
M (prevention-focused job crafting)	0.094	0.040	2.299	0.022
Constant	4.781	0.051	92.649	0.000
**Regression 3 total effect (c)**				
Predictor	Outcome = Y (performance)			
	*B*	*SE*	*t*	*p*
X (burnout)	−0.686	0.075	−9.096	0.000
Constant	4.784	0.052	91.101	0.000

B, Beta; SE, Standard error; Bootstrap sample size = 10,000.

Table 5 shows the multi-step mediation analyses. We analyze the influence of burnout on performance, including the effect of promotion-focused job crafting as a mediator. In Model 1, the burnout variable shows a significant influence on the promotion-focused job crafting mediator variable (*B* = −0.349, *SE* = 0.071, *t* = −4.863; *p* = 0.000). According to Regression 2 X, M => Y (c′ & b), it shows us the partial significant mediator effect of the promotion-focused job crafting variable, since the coefficient decreases concerning the total effect model of the independent variable when the mediator is added, the significance does not change. The Regression 3 total effect shows that the total influence of burnout on performance was significant (*B* = −0.686, *SE* = 0.075, *t* = −9.096; *p* = 0.000).

**Table 5:** Analysis of promotion-focuses job crafting mediation on the influence of burnout on performance (simple mediation model for promotion-focuses job crafting).

**Regression 1 X= > M (a)**				
Predictor	Outcome = M (promotion-focused job crafting)			
	*B*	*SE*	*t*	*p*
X (burnout)	−0.349	0.071	−4.863	0.000
Constant	0.014	0.049	−0.293	0.769
**Regression 2 X, M= > Y (c′ & b)**				
Predictor	Outcome = Y (performance)			
	*B*	*SE*	*t*	*p*
X (burnout)	−0.571	0.074	−7.675	0.000
M (promotion focuses job crafting)	0.329	0.056	5.843	0.000
Constant	4.789	0.049	96.939	0.000
**Regression 3 total effect (c)**				
Predictor	Outcome = Y (performance)			
	*B*	*SE*	*t*	*p*
X (burnout)	−0.686	0.075	−9.096	0.000
Constant	4.784	0.052	92.101	0.000

B, Beta; SE, Standard error; Bootstrap sample size = 10,000.

Table 6 shows the indirect effects (IE) of each analyzed regression. The IE2 and IE4 models are the only ones that show significance (IE2: −0.254/−0.088; IE4: −0.198/−0.051) with coefficients in terms of indirect influence of −0.167 (IE2) and − 0.115 (IE4) respectively. Its fully standardized direct effects (ab_cs_) of −0.113 (95% BC CI of −0.171 to −0.059) for IE2; and from −0.059 (95% BC CI of −0.105 to −0.022). On the other hand, Models IE1 and IE3 do not present indirect effects since they do not meet the mediation criteria.

**Table 6:** Indirect effects of the serial multiple mediator model of the effect of burnout (x) on selfefficacy (Y) and performance (Z) through prevention-focused job crafting (M1) and promotion-focused job crafting (M2).

Bootstrapping BC 95% CI				
	Coefficient	*SE*	Lower	Upper
IE1: X => M1 => Y	−0.002	0.013	−0.021	0.025
IE2: X => M2 => Y	−0.167	0.042	−0.254	−0.088
IE3: X => M1 => Z	−0.005	0.011	−0.035	0.018
IE4: X => M2 => Z	−0.115	0.037	−0.198	−0.051

IE, Indirect effect; M1, Mediator 1; M2, Mediator 2; SE, Standard error; Bootstrap sample size = 10,000.

**Figure 4:**
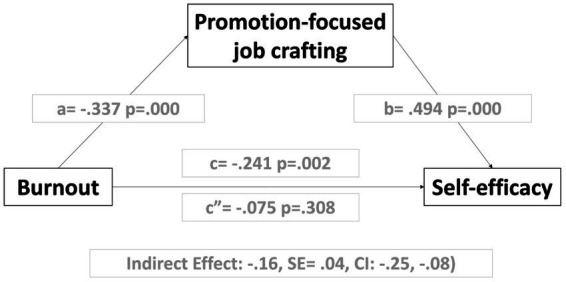
Results in mediation models of the promotion of job crafting on the influence of burnout on self-efficacy.

**Figure 5:**
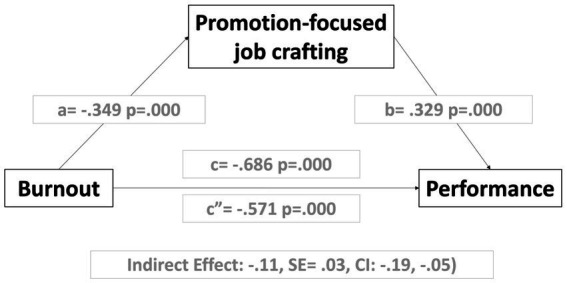
Results in mediation models of the promotion of job crafting on the influence of burnout on performance.

In summary, when we study the dimensions of job crafting as mediating elements, we find that prevention-focused job crafting does not act as a mediator between burnout and self-efficacy, nor between burnout and performance. However, when we analyze promotion, the data indicate just the opposite. Promotion-focused job crafting is a mediating variable in the relationship between burnout and self-efficacy and the relationship between burnout and performance (see Figures 4, 5).

## 4. Discussion

Throughout this work, we seek to deepen the knowledge about the concept of job crafting and its dimensions, as well as its role in the process of health deterioration proposed by JD-R theory (Bakker and Demerouti, 2013). The objective has been to analyze whether the different dimensions of job crafting mediate the influence of burnout on self-efficacy and performance. This research has been carried out based on the dimensions of existing job crafting, as proposed by Lichtenthaler and Fischbach (2019): Prevention-focuses and promotion-focuses. The objective raises the influence of burnout on prevention-focused job crafting would be positive. In promotion-focused job crafting, this influence would be negative. The results found partially support the proposed general objective. Only the promotion-focused dimension showed a mediating function in the effect of burnout on performance and self-efficacy.

The first hypothesis raised that burnout would have a positive and significant impact on prevention-focused job crafting and that it would act as a mediator in the influence of burnout on self-efficacy was refuted. Specifically, the data indicate that this job crafting dimension does not present an indirect effect. We found similar data for hypothesis 3, which stated that burnout has a positive and significant influence on the prevention of job crafting and that it would mediate the impact of burnout on performance. As there is no mediation of job crafting prevention, this hypothesis is also not confirmed.

Concerning hypotheses of the promotion-focused job crafting (hypotheses 2 and 4) were confirmed. Hypothesis 2 established that burnout has a negative and significant influence on promotion-focused job crafting and that it will be a mediator in the impact of burnout on self-efficacy. Hypothesis 4 predicted that burnout has a negative and significant effect on promotion job crafting and that it will be a mediator in the relationship of the impact of burnout on performance.

The results confirming hypotheses 2 and 4 agree with the key findings of Lichtenthaler and Fischbach (2019), who found that promotion-focused job crafting is negatively related to burnout. In addition, it represents an advance in the knowledge of how burnout acts on the behavior and perception of employees. Specifically, these data indicate that the process through which burnout negatively affects workers’ performance and self-efficacy is by reducing promotion-focused job crafting, that is, learning, achievement, personal growth, and motivational aspects. However, these workers do not use prevention strategies to protect themselves against negative states. They do not anticipate discomfort or loss of energy and health.

It can be drawn four implications from these results. First, when a worker is burning at work does not have a positive effect on his prevention responses. It can interpret that the employee gives the fact of being in a state of burnout and does not make self-protective responses. It may assume they are going through apathy, with an increasingly negative effect on the worker and on the organization. One possible explanation is the conservation or resources theory (Hobfoll, 1989). When an employee is burned out, he tries not to spend resources or protect himself.

Second, promoting job crafting is key to obtaining results and increasing employees’ personal resources. The results of this study have shown the great sensitivity of these behaviors to the negative situations that the worker experiences in his work context, highlighted by his performance and the employee’s resources. These findings support previous research that has shown the effect negative of burnout on job crafting (Yerkes and Dodson, 1908; Hobfoll, 1989; Baumeister et al., 2001; Taris et al., 2002; Lichtenthaler and Fischbach, 2019).

Third, the results confirm the negative consequences that burnout seems to exert on self-efficacy and performance. This influence is through job crafting behavior and occurs actively in the reduction of promotion-focused job crafting and passively in the absence of effect on prevention-focused job crafting. These data support previous research that has shown the negative impact of burnout on the workplace’s positive consequences (Aftab et al., 2012; Tims et al., 2014; Hosseini et al., 2017).

Fourth, promotion-focused job crafting is a mediator in the relationship between burnout, self-efficacy, and performance. Burned workers reduced their growth strategies and positive adaptation to their job.

The promotion-focused job crafting has shown a total mediation on the improvement workers’ perception of self-efficacy. In contrast, the mediation effect of this dimension of job crafting is partial to the performance. The results of this paper indicate that all the influence of burnout on self-efficacy is due to the impact of the former on promotion-focused job crafting. In contrast, the effect of burnout on performance partially depends on promotion-focused job crafting. These findings partially support the health impairment process of JD-R theory (Bakker and Demerouti, 2013).

Finally, it is important in the discussion to keep in mind that the results obtained from this research should be interpreted in relation to the characteristics of the sample. In this study, it was observed that the most representative group was comprised of individuals between 46 and 55 years of age and with a high educational level, employed in a public administration. This may imply that the levels obtained in the study variables are affected by the characteristics of the sample. However, comparing the results obtained in this work with previous studies in samples of both public administration and private entities (Mañas-Rodríguez et al., 2020; Martínez-Díaz et al., 2020, 2021; Díaz-Fúnez et al., 2021) there do not seem to be significant differences in the results of these variables.

### 4.1. Theoretical implications

This study presents two proposals for advancing scientific knowledge around the health impairment process proposed by the JD-R theory (Bakker and Demerouti, 2013) and sheds light on the spiral of health deterioration in the JD-R theory (exhaustion-job crafting-new demands influence). First, these results suggest the existence of unresolved demands, which cause burnout syndrome, and this negative emotion affects the worker’s self-protective behavior. The results found that workers will stop giving protective responses aimed at preventing the incidence of demands in their daily lives (job crafting prevention). They make the protective effect of prevention-focused job crafting disappear.

Second, the growth-oriented behaviors associated with job crafting (promotion) are reduced when burnout appears. In this situation, the adverse effects of this disorder increase. Since not only does the worker stop protecting himself through prevention actions, but there is also a lack of growth, typical of promotion actions. This situation generates a direct effect of reducing the perception of self-efficacy. At the same time, it presents a direct consequence on performance, and it is not proposed in the JD-R theory since this does not show a direct effect of job crafting on performance.

These theoretical suggestions suppose a detailed analysis of job crafting dimensions from the JD-R theory (Tims et al., 2014) and the partial refutation of the previous results of Lichtenthaler and Fischbach (2019).

Despite corroborating the negative influence of burnout on promotion job crafting, and its measuring effect on self-efficacy and performance. When workers are burnout, they do not show an impact on prevention-focused job crafting, cutting off this pathway of influence on self-efficacy and performance.

### 4.2. Practical implications

Employee burnout at work negatively affects performance and self-efficacy. Organizations must prevent their employees from reaching this situation, both due to the adverse effects on the development of the employee and the organization itself and due to the apathy generated in the worker to reduce the rest of the demands. For example, a burned-out worker stops growing professionally but is also more affected by the new demands in the work context by not carrying out prevention behaviors. Establishing the necessary corrective measures to prevent this syndrome from appearing is key.

Another practical implication is the central role of promotion-focused job crafting in public management. In this sense, if it wants to prevent burnout in this work context, another way is to offer promotion strategies. For example, it can promote actions of growth, learning, and career development of employees. These findings lead us to value professional careers as a strategy to prevent employee burnout in the workplace.

### 4.3. Limitations and future research

However, the results obtained in this study must be considered under three limitations. Firstly, the results were obtained from online self-reports and could be affected by common method variance. However, the results of the Harman test (Podsakoff et al., 2003) showed that the exploratory factor analysis with all the study variables produced values in the first factor that did not exceed 50% of the variance between the variables (46.2%; Podsakoof and Organ, 1986). Furthermore, a poor model fit was revealed (X2 = 11231.553, *p* < 0.001), which means that common method variation would not be a serious deficiency in this study.

Second, the sample is very specific, limited to the group of administration and service personnel of a public administration. Therefore, the results should be generalized to other types of organizations with caution. However, the results are interesting as inputs for interventions to improve employee well-being and develop healthy public organizations.

Third, the study design is cross-sectional, which prevents conclusions from being drawn about the temporal order of effects and causal relationships. However, the longitudinal effects of the test were not the main objective of this study, since we tested a mediation model of job crafting dimensions in employees.

Following the above limitations, we suggest other forms of data collection using records obtained through direct observation or critical incident assessment interviews. This would provide complementary measures to corroborate the goodness of the data used.

Second, it might be convenient to increase the sample spectrum of the study (e.g., compare samples from public and private administration) for a multivariate investigation. Longitudinal studies are needed to analyze the evolution and causal influences on the health impairment process by JD-R theory.

Finally, other variables could be incorporated in future studies to have extended models. For this, leadership style could be a critical variable. It is well known that the behavior of leaders has an important influence on employees. Consequently, different leadership styles (e.g., transformational) could be investigated as moderators of the effect of burnout on job crafting dimensions. Another relevant variable may be the organizational climate or culture. Authors such as Wei et al. (2021) have already shown how this variable adds valuable information to understand the impact of different perceptions of the organizational environment of public employees.

## 5. Conclusion

The results indicate the high sensitivity of job crafting behaviors (both prevention-focused and promotion-focused) in situations of employee exhaustion, such as burnout syndrome. Specifically, this study presents how the absence of prevention responses and the negative effect on promotion behaviors are two of the ways through which burnout negatively influences the organization and employees.

## Data availability statement

The raw data supporting the conclusions of this article will be made available by the authors, without undue reservation.

## Ethics statement

The studies involving human participants were reviewed and approved by University of Almeria Bioethics committee. The patients/participants provided their written informed consent to participate in this study.

## Author contributions

AM-D, PD-F, and MM-R contributed to conception and design of the study. BH-S and JS-G organized the database. BH-S and PD-F performed the statistical analysis. PD-F, MM-R, and JS-G wrote the first draft of the manuscript. AM-D, CS-F, BH-S, and PD-F wrote sections of the manuscript. All authors contributed to the article and approved the submitted version.

## Conflict of interest

The authors declare that the research was conducted in the absence of any commercial or financial relationships that could be construed as a potential conflict of interest.

## Publisher’s note

All claims expressed in this article are solely those of the authors and do not necessarily represent those of their affiliated organizations, or those of the publisher, the editors and the reviewers. Any product that may be evaluated in this article, or claim that may be made by its manufacturer, is not guaranteed or endorsed by the publisher.
